# Beyond the Brain: A Case of Metastatic Astrocytoma

**DOI:** 10.7759/cureus.117920

**Published:** 2026-10-14

**Authors:** Ana David do Carmo, Beatriz Tallon, Filipa Malheiro

**Affiliations:** 1 Department of Internal Medicine, Hospital da Luz Lisboa, Lisbon, PRT

**Keywords:** grade 4 astrocytoma, isocitrate dehydrogenase-mutated, metastatic astrocytoma, metastatic brain tumors, supportive and palliative care

## Abstract

Astrocytomas are primary brain tumors characterized by aggressive growth and local invasion. They typically remain confined to the central nervous system (CNS), and distant metastases are extremely uncommon.

We present the case of a 45-year-old man diagnosed with an isocitrate dehydrogenase (IDH)-mutant, grade 4 astrocytoma (World Health Organization (WHO) classification), who underwent gross total resection followed by adjuvant chemoradiotherapy, initially showing good clinical progress. After the fourth cycle of adjuvant temozolomide, he developed lower back pain, asthenia, and sweating that led to further investigation revealing extensive bone infiltration and an epidural paravertebral mass at the second lumbar vertebra (L2), which was histologically confirmed as bone metastasis with epidural extension and bone marrow infiltration by the astrocytoma. Due to the lack of eligibility for further curative therapies, and after multiple hospitalizations for symptom management, the patient was admitted to the Palliative Care Unit, where he ultimately passed away.

This case adds to the clinical knowledge by documenting a rare presentation of IDH-mutant astrocytoma with extracranial bone metastases, a phenomenon seldom seen in gliomas. It highlights the diagnostic complexity of distinguishing between treatment-related effects and disease progression when gliomas present with systemic symptoms. Additionally, it underscores the challenges of managing advanced disease with limited treatment options and emphasizes the role of palliative care in symptom control.

## Introduction

Central nervous system (CNS) tumors are less common than many other cancers but represent the leading cause of cancer-related death in individuals under the age of 40 [1,2]. Western Europe has among the highest incidence rates of brain and CNS cancers worldwide, with an estimated age-standardized rate of approximately 5.6 cases per 100,000 person-years in 2022, comparable to that of North America [3]. Gliomas account for approximately 30% of all primary brain tumors and about 80% of all malignant brain tumors [4,5]. In Europe, astrocytic tumors represent the large majority of malignant CNS cancers [4].

Astrocytomas are a group of primary brain tumors arising from astrocytic glial cells and represent a significant proportion of adult diffuse gliomas [6]. According to the World Health Organization (WHO) classification of CNS tumors, astrocytomas are graded from 2 to 4 based on histological and molecular features, with higher grades associated with more aggressive clinical behavior and poorer prognosis [7-9]. Diffuse astrocytomas occur predominantly in adults, with peak incidence between the fourth and sixth decades of life, and are slightly more common in men than women [10-12]. The overall incidence of isocitrate dehydrogenase (IDH)-mutant astrocytomas is estimated at approximately 1.2 cases per 100,000 persons per year, with geographic and ethnic variation reported, being highest in populations of European ancestry and lower in Black and Asian populations [2,6,13].

Despite their locally aggressive nature, astrocytomas rarely metastasize outside the CNS. The incidence of extracranial metastases from diffuse gliomas is estimated to be below 2%, likely due to several factors including the absence of lymphatic drainage in the brain, the presence of the blood-brain barrier, and the typically short survival of affected patients [14]. When metastasis occurs, it most frequently involves the regional lymph nodes, lungs, and pleura, and less frequently bone and liver [15].

Several factors have been proposed to increase the risk of extracranial dissemination, including prior neurosurgical interventions, ventriculoperitoneal shunting, radiotherapy, and prolonged survival that allows time for metastatic spread to develop [14,16]. Although metastases have historically been associated more commonly with IDH-wildtype glioblastomas, reports involving IDH-mutant astrocytomas have increasingly emerged, particularly in patients receiving multimodal treatment and achieving longer survival [17]. Patients with extracranial metastases generally have a poor prognosis, with reported median survival after detection of metastasis ranging from a few months to approximately one year [14,18].

In this report, we present the case of a 45-year-old male patient diagnosed with an IDH-mutant, WHO grade 4 astrocytoma who, despite undergoing standard multimodal therapy, developed extensive bone metastases with epidural extension and bone marrow infiltration. This case highlights the diagnostic and therapeutic challenges associated with glioma dissemination beyond the CNS and underscores the importance of clinical awareness and multidisciplinary management in cases of atypical disease progression.

## Case presentation

A 45-year-old male patient with a past medical history of arterial hypertension, dyslipidemia, and atrial fibrillation treated with catheter ablation one month earlier presented to the emergency department with a two-month history of headache localized in the occipital region, left hemianopsia, dysmetria, and ataxia predominantly affecting the left upper limb. These neurological symptoms developed progressively over this two-month period.

The patient had no prior personal history of CNS tumors or other malignancies. Family history was notable for breast cancer in his mother and maternal aunt, lung adenocarcinoma in his father, and colon cancer in his paternal grandfather. There was no personal history of prior cranial irradiation, and no clinical features suggestive of a hereditary cancer-predisposition syndrome were present. Before symptom onset, he was fully independent in activities of daily living, with an Eastern Cooperative Oncology Group (ECOG) performance status of 0.

During the etiological investigation, cranial computed tomography (CT) and magnetic resonance imaging (MRI) revealed a heterogeneous multicystic expansive lesion in the right parietal region (Figure 1). The patient underwent gross total resection.

**Figure 1:**
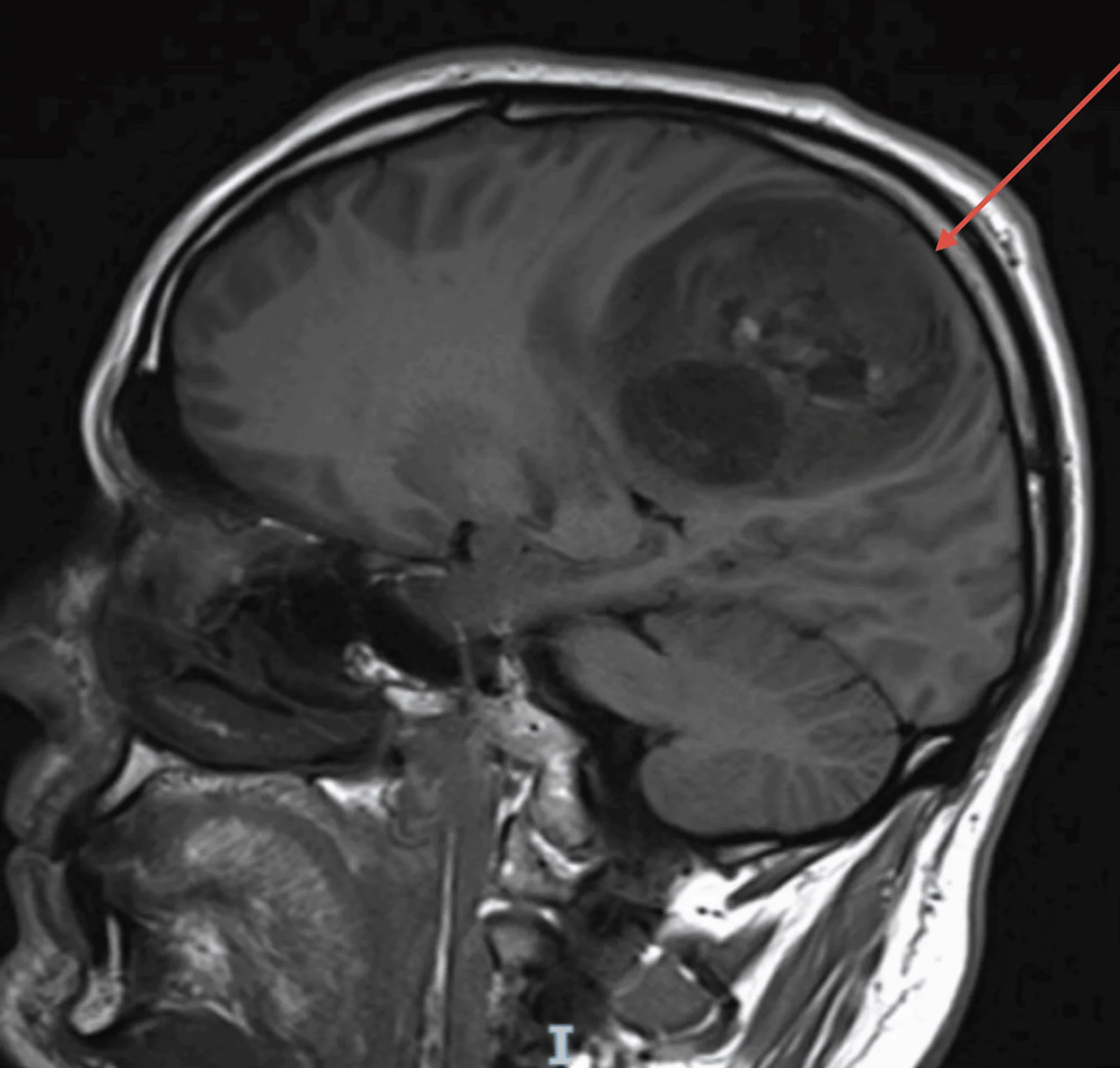
Magnetic resonance imaging (MRI) of the brain revealing a heterogeneous multicystic expansive lesion in the right parietal region (red arrow)

Histopathological examination revealed an infiltrative glial neoplasm with astrocytic morphology, showing marked cellular pleomorphism with multinucleated giant cells and small anaplastic cells. Mitotic activity, microvascular proliferation, and areas of necrosis were observed. Immunohistochemical analysis showed glial fibrillary acidic protein (GFAP) positivity, IDH1 R132H mutation, loss of alpha-thalassemia/mental retardation syndrome X-linked (ATRX) expression, negative p53 expression, variable synaptophysin expression, preserved H3K27me3 expression, and a high proliferative index (Ki-67/MIB-1). 1p/19q codeletion status was not assessed, as loss of ATRX expression in an IDH-mutant glioma is virtually mutually exclusive with 1p/19q codeletion and supports the diagnosis of astrocytoma. O6-methylguanine-DNA methyltransferase (MGMT) promoter methylation status was also not assessed. These findings were consistent with astrocytoma, IDH-mutant, WHO CNS grade 4 (WHO 2021 classification).

Postoperatively, the patient was treated according to the Stupp et al. protocol [19], which consisted of focal radiotherapy (60 Gy in 30 fractions) with concurrent daily temozolomide (75 mg/m²), followed by adjuvant temozolomide (150 mg/m² on days 1 to 5 of each 28-day cycle). After completing the concurrent phase, the patient was neurologically asymptomatic. A brain MRI performed four weeks later showed postoperative changes and a small enhancing focus suggestive of minimal residual tumor, without new intracranial lesions, and adjuvant temozolomide was then started.

Following completion of the fourth cycle of adjuvant temozolomide, the patient presented to the emergency department on several occasions with mechanical lower back pain and retrosternal pain, with associated fatigue and sweating, without fever, dyspnea, or weight loss. Physical examination, including neurological assessment of the lower limbs, was unremarkable.

Laboratory findings revealed markedly elevated D-dimers, normal high-sensitivity troponin, leukopenia, thrombocytopenia, elevated C-reactive protein (CRP) and alkaline phosphatase (ALP), and markedly increased lactate dehydrogenase (LDH), as shown in Table 1.

**Table 1:** Laboratory findings with corresponding reference values CRP: C-reactive protein; ALP: alkaline phosphatase; LDH: lactate dehydrogenase; IU: international units

Laboratory parameter	Patient value	Reference value
D-dimers	15.47 mg/L	<0.5 mg/L
High-sensitivity troponin	<4 ng/L	<58 ng/L
Leukocyte count	2.66 × 10⁹/L	3.70-9.50 × 10⁹/L
Platelet count	94 × 10⁹/L	170-430 × 10⁹/L
CRP	19 mg/dL	<0.5 mg/dL
ALP	307 IU/L	46-116 IU/L
LDH	2,513 IU/L	85-227 IU/L

A thoracic CT angiography ruled out pulmonary embolism. A lumbar MRI identified extensive bone infiltration by lesions of probable secondary origin, with a paravertebral and epidural mass at the second lumbar vertebra (L2) compressing the thecal sac and likely causing nerve root compression (Figure 2).

**Figure 2:**
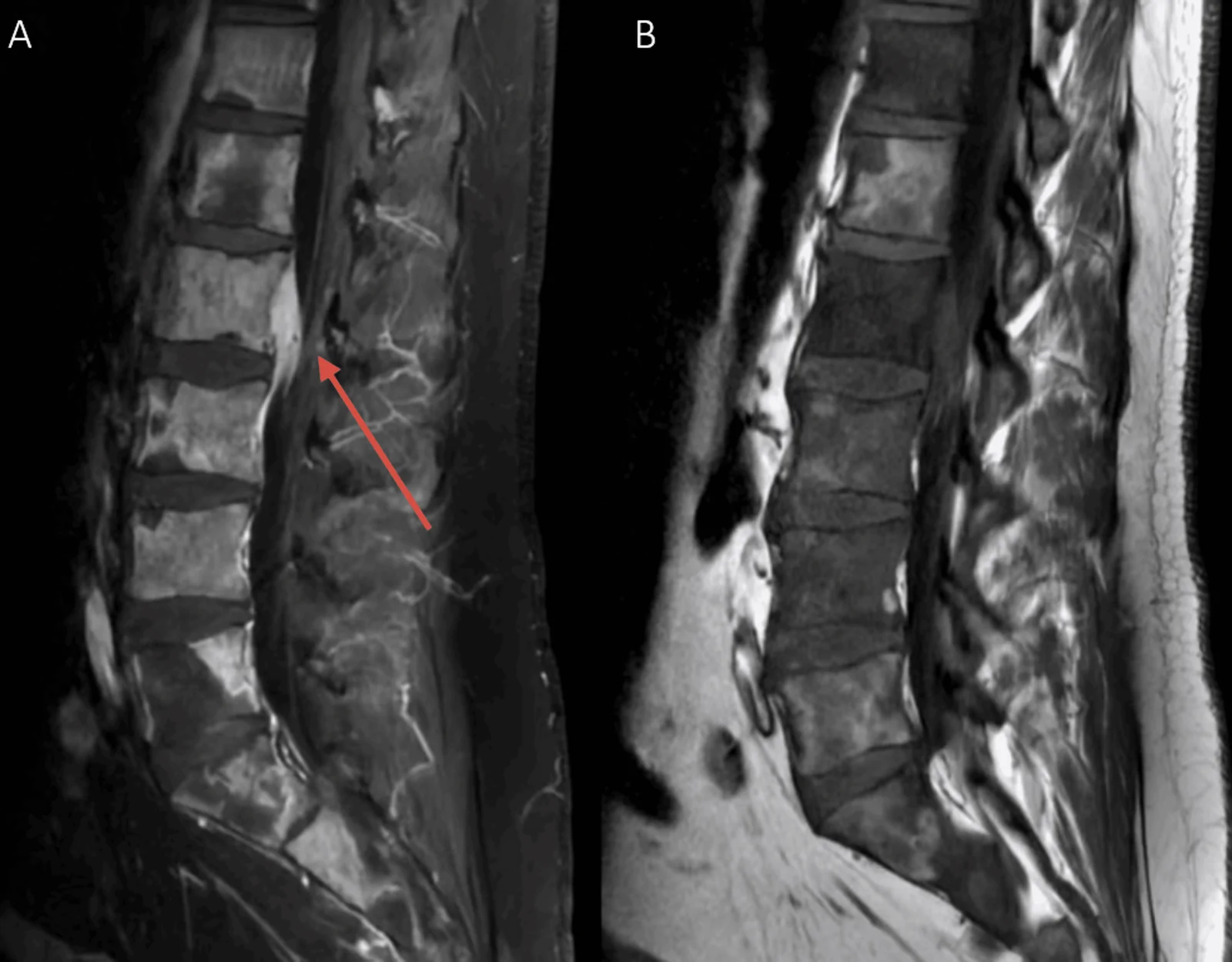
(A, B) Two magnetic resonance imaging (MRI) images of the lumbar spine demonstrating extensive infiltrative lesions with a paravertebral and epidural mass causing thecal sac compression (red arrow)

Bone marrow aspiration and biopsy, together with biopsy of the bone lesion, were performed. Immunocytochemical and immunohistochemical analysis of these samples showed expression of GFAP, oligodendrocyte transcription factor 2 (OLIG2), and IDH1 R132H, confirming bone metastasis and bone marrow infiltration by the IDH-mutant astrocytoma (Figure 3).

**Figure 3:**
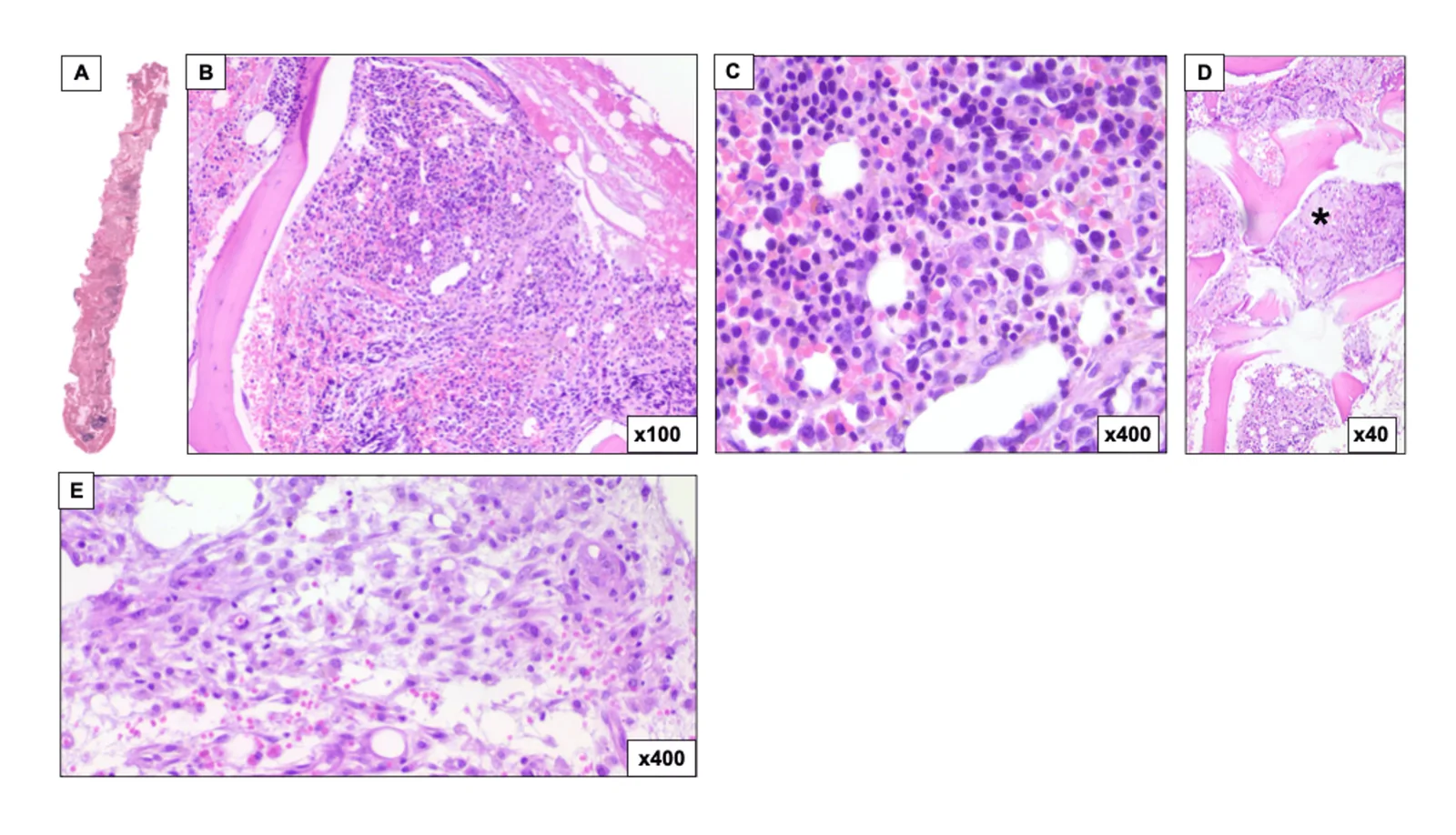
Bone marrow core needle biopsy (hematoxylin and eosin staining) (A) Low-power view of the bone marrow core needle biopsy. (B, 100×) Marrow lesion composed of neoplastic cells arranged in sheets, with (C, 400×) eosinophilic cytoplasm and eccentric nuclei, and associated (D, 40×) necrosis (asterisk) and (E, 400×) numerous tumor-associated macrophages.

Following the histopathological diagnosis, TP53 gene testing in peripheral blood was proposed to exclude a possible germline origin, but it could not be performed due to the subsequent rapid disease progression. For staging purposes, a whole-body CT scan was performed, which showed no evidence of metastases at other sites.

In light of the confirmed disease progression and the severity of the cytopenias, temozolomide was permanently discontinued. The disease progressed with severe pancytopenia (minimum values: hemoglobin 5.8 g/dL, leukocytes 0.17 × 10⁹/L, platelets 4 × 10⁹/L), requiring multiple blood product transfusions.

After multidisciplinary discussion, the patient underwent five sessions of palliative radiotherapy targeting the bone lesions and the paravertebral and epidural mass for nerve root compression control. Other systemic therapies were not considered appropriate given the confirmed disease progression and the severity of pancytopenia. Following multiple hospitalizations for symptom management, the patient ultimately passed away in the Palliative Care Unit, 53 days after completion of palliative radiotherapy.

## Discussion

The present case describes an IDH-mutant, WHO grade 4 astrocytoma with extracranial dissemination to bone and bone marrow, diagnosed during adjuvant temozolomide. Three features make it particularly unusual: the rarity of extracranial metastasis in gliomas, especially in IDH-mutant tumors; the involvement of the bone marrow, which is even more rarely described; and the early onset of metastatic disease compared with the course typically reported in the literature. To place these findings in context, the etiology, classification, and biology of IDH-mutant astrocytomas are first reviewed.

Although the etiology of most gliomas remains unclear, the most well-established risk factor is prior exposure to ionizing radiation to the brain [10]. Certain genetic cancer predisposition syndromes, including neurofibromatosis type 1, Li-Fraumeni syndrome, Turcot syndrome, and Lynch syndrome, have also been associated with an increased risk of glioma development [20,21]. In the present case, TP53 gene testing in peripheral blood was proposed to exclude a germline origin, such as Li-Fraumeni syndrome, but could not be performed. Nevertheless, the majority of gliomas occur sporadically without identifiable predisposing factors [22].

The 2021 WHO classification for CNS tumors adopts a framework combining histological and molecular information (such as genetic mutations and chromosomal alterations). The classification for diffuse gliomas in adults is summarized in Table 2 [23].

**Table 2:** Molecular classification of adult-type diffuse gliomas according to the WHO CNS 2021 classification Diagnostic algorithm for the molecular classification of adult-type diffuse gliomas according to the 2021 WHO Classification of Tumors of the Central Nervous System [23]. Diffuse gliomas are first stratified according to IDH mutation status. IDH-mutant tumors are further classified based on 1p/19q codeletion status, whereas IDH-wildtype tumors are diagnosed as glioblastoma if they harbor characteristic molecular features. IDH: isocitrate dehydrogenase; EGFR: epidermal growth factor receptor; TERT: telomerase reverse transcriptase; WHO: World Health Organization; CNS: central nervous system

Step	Molecular feature	Resulting tumor type	WHO grade
1	IDH mutation status		
	IDH-mutant	Proceed to 1p/19q codeletion testing	-
	IDH-wildtype	Evaluate glioblastoma molecular features	-
2	1p/19q codeletion (in IDH-mutant tumors)		
	Present	Oligodendroglioma, IDH-mutant and 1p/19q-codeleted	Grade 2-3
	Absent	Astrocytoma, IDH-mutant	Grade 2-4
3	Glioblastoma molecular features (in IDH-wildtype tumors)		
	Microvascular proliferation or necrosis, or EGFR amplification, or TERT promoter mutation, or chromosome +7/−10 signature	Glioblastoma, IDH-wildtype	Grade 4

Histopathologically, astrocytomas are characterized by infiltrative growth of neoplastic astrocytic cells with varying degrees of cellular pleomorphism, mitotic activity, microvascular proliferation, and necrosis in higher-grade tumors [24]. Immunohistochemical markers such as GFAP, IDH1 mutation, and loss of ATRX expression support astrocytic lineage and molecular classification [25]. In the present case, the tumor demonstrated features consistent with an infiltrative astrocytic neoplasm with cellular pleomorphism, microvascular proliferation, necrosis, and a high proliferative index, confirming the diagnosis of IDH-mutant astrocytoma, WHO CNS grade 4. Metastatic involvement of bone and bone marrow was confirmed by histopathological analysis, with both the bone lesion and the bone marrow samples showing expression of GFAP, OLIG2, and IDH1 R132H, consistent with the primary tumor. Radiologically, the primary lesion appeared as a heterogeneous multicystic mass, in keeping with a high-grade tumor, whereas the extracranial lesions had a nonspecific imaging appearance and required histological confirmation to establish their glial origin.

Advances in molecular diagnostics, particularly the identification of IDH mutations, have introduced new complexity to the clinical behavior of these tumors. IDH-mutant gliomas are generally associated with a better prognosis compared to IDH-wildtype gliomas [17,26]. This is because IDH mutations lead to the production of an oncometabolite, 2-hydroxyglutarate, which interferes with cellular metabolism and epigenetic regulation. While this mutation drives tumorigenesis, IDH-mutant gliomas tend to exhibit slower growth, less aggressive behavior, and a lower propensity for metastatic spread. The improved prognosis in IDH-mutant gliomas is also reflected in their response to therapies, such as chemotherapy and radiation [26-29]. This is in contrast to IDH-wildtype gliomas, particularly glioblastomas, which are more aggressive, have a higher recurrence rate, and are less responsive to conventional therapies [17]. Extracranial dissemination is therefore particularly unexpected in IDH-mutant tumors, as observed in the present case.

Among extracranial sites, bone marrow involvement is particularly rarely described. Moreover, extraneural metastases from gliomas typically occur late in the course of the disease, at a median of approximately two years after diagnosis [15], whereas in the present case, they were detected within the first year after diagnosis.

The brain's unique microenvironment, which includes the blood-brain barrier, plays a crucial role in the relatively low incidence of glioma metastasis beyond the brain. While the mechanism behind extracranial metastases remains unclear, it is hypothesized that disruption of the blood-brain barrier due to prior surgical or radiotherapeutic interventions may allow tumor cells to enter the systemic circulation [14]. In this case, the development of bone metastasis and bone marrow invasion after standard treatment, including surgery, radiotherapy, and temozolomide, supports that treatment-related disruption of the blood-brain barrier may have facilitated the spread of tumor cells, leading to extracranial metastasis.

The pancytopenia observed raised an initial differential between temozolomide-related myelosuppression, a recognized dose-limiting toxicity of the drug, and bone marrow infiltration by the underlying malignancy [30]. The concurrent lower back and retrosternal pain, however, could not be attributed to treatment-related toxicity, as the radiotherapy field had been restricted to the cranium. This prompted an independent imaging workup, which identified extensive skeletal infiltration and confirmed that both the pain and the cytopenias shared a common cause: bone marrow and skeletal involvement by the astrocytoma.

Treatment of high-grade astrocytomas typically involves a multimodal approach combining maximal safe surgical resection followed by radiotherapy and chemotherapy. The current standard of care is the Stupp protocol, which consists of radiotherapy administered concurrently with temozolomide followed by adjuvant temozolomide cycles [19]. This combination significantly improves survival compared with radiotherapy alone, with a median overall survival of 14.6 versus 12.1 months and a two-year survival rate of 27.2% versus 10.9%, a benefit that persisted at five years (9.8% versus 1.9%) [19,31]. These trials enrolled patients with glioblastoma before the introduction of molecular classification, and the protocol has since been extended to IDH-mutant grade 4 astrocytomas.

When temozolomide is unavailable, not tolerated, or in cases of disease progression, nitrosourea-based regimens such as procarbazine, lomustine, and vincristine have historically been used as an alternative in high-grade gliomas [32]. The clinical management of extracranial metastases from gliomas is challenging due to the limited efficacy of systemic therapies. In this case, palliative radiotherapy was employed to alleviate symptoms related to thecal sac and nerve root compression. Despite these efforts, the disease progressed, leading to severe pancytopenia that required multiple transfusions, reflecting both the burden of marrow invasion and the limitations of supportive care in advanced disease stages. The patient died 53 days after completion of palliative radiotherapy, consistent with the poor prognosis reported after detection of extracranial metastasis [14,18].

Comparable cases of IDH-mutant astrocytoma with extracranial metastases have been reported only sporadically. The case most similar to ours described a 32-year-old man with a recurrent grade 4 IDH-mutant astrocytoma who developed lumbar vertebral lesions, pancytopenia, and a progressive rise in LDH, with bone marrow infiltration confirmed on biopsy. He received palliative radiotherapy and died shortly after the diagnosis of diffuse bone marrow metastasis, and the authors proposed that progressive LDH elevation may herald bone marrow involvement in patients with high-grade glioma [33]. Other reports have described bone metastases in young adults with grade 4 IDH-mutant astrocytomas, in some cases accompanied or preceded by lymph node or leptomeningeal involvement, including tumors that had progressed from lower-grade gliomas [34-36]. In one of these cases, as in ours, adjuvant temozolomide had to be discontinued because of pancytopenia, although the patient survived approximately one year after the first extracranial metastasis, illustrating the variable course of this condition [35].

In these reports, management of extracranial disease was largely palliative, consisting mainly of local radiotherapy and supportive care, with second-line chemotherapy attempted in selected patients but limited by hematological toxicity [33-35]. Taken together with the present case, these reports support considering extracranial dissemination when patients with high-grade gliomas develop bone pain, unexplained cytopenias, or rising LDH.

Routine systemic staging is not recommended in patients with gliomas given the rarity of extracranial spread [5], and there is no evidence that earlier detection or an alternative treatment protocol would have changed the outcome in this case. Nevertheless, new bone pain, unexplained cytopenias, or markedly elevated LDH during or after treatment should prompt consideration of extracranial metastasis, including bone marrow examination, rather than being attributed solely to treatment toxicity.

## Conclusions

This case report presents a rare instance of IDH-mutant astrocytoma with extracranial bone and bone marrow metastases with epidural extension, a phenomenon scarcely observed in gliomas. Despite standard treatment, including gross total resection and radiotherapy with concurrent temozolomide, the patient unexpectedly developed metastatic disease during adjuvant temozolomide. While metastases in gliomas are uncommon, disruption of the blood-brain barrier due to surgical and radiotherapeutic interventions may facilitate the rare spread of tumor cells to distant sites. Given the limited therapeutic options for advanced gliomas with metastatic spread, the role of palliative care becomes crucial in managing symptoms and improving quality of life.

In patients with high-grade gliomas, new bone pain, unexplained cytopenias, or rising LDH should prompt consideration of extracranial metastasis. This case contributes to the understanding of glioma's potential for distant metastasis and emphasizes the importance of a multidisciplinary approach in the management of such complex cases.
